# Detection of Indiscriminate Genetic Manipulation in Thoroughbred Racehorses by Targeted Resequencing for Gene-Doping Control

**DOI:** 10.3390/genes13091589

**Published:** 2022-09-04

**Authors:** Teruaki Tozaki, Aoi Ohnuma, Kotono Nakamura, Kazuki Hano, Masaki Takasu, Yuji Takahashi, Norihisa Tamura, Fumio Sato, Kyo Shimizu, Mio Kikuchi, Taichiro Ishige, Hironaga Kakoi, Kei-ichi Hirota, Natasha A. Hamilton, Shun-ichi Nagata

**Affiliations:** 1 Genetic Analysis Department, Laboratory of Racing Chemistry, 1731-2, Tsurutamachi, Utsunomiya 320-0851, Japan; 2 Department of Veterinary Medicine, Faculty of Applied Biological Sciences, Gifu University, 1-1, Yanagido, Gifu 501-1193, Japan; 3 Equine Research Institute, Japan Racing Association, 1400-4, Shiba, Shimotsuke 329-0412, Japan; 4 Registration Department, Japan Association for International Racing and Stud Book, 4-5-4, Shimbashi, Minato, Tokyo 105-0004, Japan; 5 Equine Genetics Research Centre, Racing Australia, 2 Randwick Way, Scone, NSW 2337, Australia

**Keywords:** amplicon sequencing, gene doping, gene editing, horse, thoroughbred

## Abstract

The creation of genetically modified horses is prohibited in horse racing as it falls under the banner of gene doping. In this study, we developed a test to detect gene editing based on amplicon sequencing using next-generation sequencing (NGS). We designed 1012 amplicons to target 52 genes (481 exons) and 147 single-nucleotide variants (SNVs). NGS analyses showed that 97.7% of the targeted exons were sequenced to sufficient coverage (depth > 50) for calling variants. The targets of artificial editing were defined as homozygous alternative (HomoALT) and compound heterozygous alternative (ALT1/ALT2) insertion/deletion (INDEL) mutations in this study. Four models of gene editing (three homoALT with 1-bp insertions, one REF/ALT with 77-bp deletion) were constructed by editing the *myostatin* gene in horse fibroblasts using CRISPR/Cas9. The edited cells and 101 samples from thoroughbred horses were screened using the developed test, which was capable of identifying the three homoALT cells containing 1-bp insertions. Furthermore, 147 SNVs were investigated for their utility in confirming biological parentage. Of these, 120 SNVs were amenable to consistent and accurate genotyping. Surrogate (nonbiological) dams were excluded by 9.8 SNVs on average, indicating that the 120 SNV could be used to detect foals that have been produced by somatic cloning or embryo transfer, two practices that are prohibited in thoroughbred racing and breeding. These results indicate that gene-editing tests that include variant calling and SNV genotyping are useful to identify genetically modified racehorses.

## 1. Introduction

Gene doping is a prohibited practice in both human and horse sports to maintain integrity [1]. The global leader of thoroughbred racing, the International Federation of Horseracing Authorities (IFHA, https://www.ifhaonline.org/, accessed on 1 September 2022), prohibits the administration of genetic materials including transgenes, therapeutic oligonucleotides, and genetically modified cells to horses; further, the IFHA and the International Stud Book Committee (ISBC, https://www.internationalstudbook.com/, accessed on 1 September 2022) prohibit the creation of genetically engineered racehorses. Genetically engineered animals (including embryos) have been recently created in many species, including horses [2,3].

The methods for creating genetically modified animals include the introduction of an exogenous gene into the host genome using transposons [4], retroviruses (including lentiviruses) [5], cell-mediated transgenesis [6], and gene editing (homologous recombination [HR] of CRISPR/Cas9) [7,8]. Gene insertion is used in gene therapy to supplement the functions of defective genes [9]. Alternatively, CRISPR/Cas9 can be used to specifically introduce short insertions or deletions (INDELs) to targeted regions via nonhomologous end joining (NHEJ) [10,11]. This facilitates gene knockdown or knockout by introducing a frameshift into the targeted gene.

Pathological models of animals using gene-editing techniques to study diseases and livestock with modified genes related to economic traits have also been developed [12,13]. Gene-editing techniques make it theoretically possible to easily perform illegal gene doping to produce genetically modified racehorses, which pose a threat to the integrity of both the horse racing industry and equestrian sports.

Many methods to detect inserted transgenes using quantitative PCR have been developed for gene-doping control [14,15,16,17,18,19,20,21,22,23]. By designing hydrolysis probes targeting the exon/exon junction, transgenes can be detected in a target-specific manner. In recent years, next-generation sequencing (NGS) technology has enabled collection of large amounts of massive parallel sequence (MPS) information. NGS technology has also enabled whole-genome resequencing of species with reference genome sequences [24,25,26]. Using NGS, transgenes can be detected as deletions of introns [27,28,29], enabling screening for nontargeted transgenes by comparing the detected intron deletions to annotated gene information (i.e., the intron location in reference sequences). Amplicon sequencing to detect targeted genes using NGS has also been developed for transgene detection [30,31].

Currently, there is no method to detect genome-edited sequences. NGS technology has enabled large-scale whole-genome resequencing (WGR), whole-exome resequencing (WER), and targeted gene or exon resequencing of species with reference genome sequences. WGR and WER are generally used to search for mutations causative of hereditary diseases [32,33], and targeted resequencing is often used to detect mutations in genes associated with cancer [34].

In this study, we developed and validated a method to identify genome-edited sequences using targeted resequencing and examined its applicability by screening gene-edited equine cells. We also evaluated the effectiveness of a single-nucleotide variant (SNV) panel to detect foals that have been produced using embryo transfer and somatic cell nuclear transfer, two artificial breeding techniques that are also prohibited in the thoroughbred racing and breeding industry.

## 2. Materials and Methods

### 2.1. Animal Ethics and Sample Collection

Blood and hair sample collection was approved by the Animal Care Committee of the Laboratory of Racing Chemistry (approval number 20-4, 13 February 2020). Samples were collected from horses at the Hidaka Training and Research Center (HTRC) of the Japan Racing Association (JRA), Japan Bloodhorse Breeders’ Association (JBBA), and donated by individual horse owners by the Japan Association for International Racing and Stud Book (JAIRS).

Hair roots (*n* = 46) and whole blood (*n* = 50) were collected from thoroughbred horses (1–15 years old) as a validation set of samples for the developed gene-editing test. Hair roots from 120 thoroughbred horses (<1 years old) were collected as casework examples to test the utility of the parent verification SNV panel.

Blood was collected in BD Vacutainer^®^ spray-coated K2EDTA tubes (Becton, Dickinson and Company, Franklin Lakes, NJ, USA). Hair with roots were pulled from the mane. Blood and hair were stored at −30 °C and 4 °C, respectively, until use.

### 2.2. DNA Extraction

Genomic DNA was extracted from whole blood (200 μL) and hair (5 and 10 roots) using a DNeasy Blood & Tissue Kit (Qiagen, Hilden, Germany). Extracted DNA was quantified using a Qubit dsDNA HS Assay Kit (Thermo Fisher Scientific, Waltham, MA, USA). The extracts were diluted to 4 ng/μL with Milli-Q.

### 2.3. Construction of Genome Edited Cells

Ear tissues of necropsied horses were provided by the Equine Research Institute, Japan Racing Association (ERI2021-711, 12 October 2021). Fibroblasts were isolated from ear tissue using an explant culture method. The tissue was finely diced (5–10 mm-thickness) using two scalpels and then cultured at 37 °C in Dulbecco’s modified Eagle’s medium (Sigma-Aldrich, St. Louis, MO, USA) containing 10% fetal bovine serum (Gibco, Grand Island, NY, USA) and gentamicin (20 μg/mL). After cell migration was confirmed, the tissues were removed from the culture medium. Primary cells were passaged once and then harvested for transfection.

The Guide-it CRISPR/Cas9 System (Takara Bio Inc., Kusatsu, Shiga, Japan) was used to edit the *myostatin* (*MSTN*) gene. gRNAs (Guide RNAs) were designed to target locations 66609921-66609940 (5′-TTTCCAGGCGCAGTTTACTG-3′) on exon 1, 66607737-66607756 (5′-TACCTTGTACCGTCTTTCAT-3′) on exon 2, and 66605475-66605494 (5′-AAATCTCTTCTGGATCGTTT-3′) on exon 3 in chromosome 18 of EquCab3.0 (GCA_002863925.1). DNA oligonucleotides of each gRNA sequence were artificially synthesized (Fasmac Co., Ltd., Atsugi, Kanagawa, Japan) and cloned into the pGuide-it plasmid vector, which contained the U6 promoter and sgRNA scaffold to transcribe gRNA, genes encoding Cas9, and green fluorescent protein (GFP).

The constructed pGuide-it vector was electroporated into cultured horse fibroblasts using the Neon^®^ Transfection System (Thermo Fisher Scientific). Transgenic cells expressing GFP were selected and a single transgenic cell line representing each edit was isolated in a 24-well culture plate (Corning, Inc., Corning, NY, USA) using the TransferMan^®^ Nk-2 Micromanipulation System (Eppendorf AG, Hamburg, Germany).

As some genome edits did not develop into sufficient numbers of cells, whole-genome amplification (WGA) was performed using the REPLI Single Cell Kit (Qiagen) as required.

Finally, genomic DNA was extracted from genome-edited cells or their WGA products using a DNeasy Blood and Tissue Kit (Qiagen). The extracted DNA was quantified using the Qubit dsDNA HS Assay Kit (Thermo Fisher Scientific). The extracts were diluted to approximately 4 ng/μL using Milli-Q.

### 2.4. Assay Design for Targeted Genes and SNVs

Fifty-two equine genes (Table 1) selected based on their known functions in several species (primarily horse, human, and mouse) and 147 single-nucleotide variants (SNVs) typically used for DNA typing were targeted in this study [35,36]. Primers for PCR amplification of targeted resequencing were designed by Illumina Concierge using DesignStudio Sequencing Assay Designer (Illumina Inc., San Diego, CA, USA) based on the horse reference genome, EquCab3.0 (GenBank: GCA_002863925.1). Each amplicon ranged from 125 base pairs (bp) to 275 bp in length.

### 2.5. Library Preparation and Sequencing

Libraries were prepared using AmpliSeq Library PLUS for Illumina (Illumina Inc.) based on the manufacturer’s recommendations. AmpliSeq CD Indexes Set A-D for Illumina (384 Indexes, Illumina Inc.) were used to index the samples. Sequencing was performed on the NextSeq 500 sequencing platform (Illumina Inc.) using the NextSeq 500/550 Mid Output Kit v2.5 (300 Cycles, Illumina Inc.).

### 2.6. Data Analysis, Variant Calling, and SNV Genotyping

Variant detection was performed using the RESEQ pipeline (Amelieff Co., Minato, Tokyo, Japan), which was constructed based on QCleaner (Amelieff Co.), Burrows-Wheeler aligner (BWA, version 0.7.17) [37], Genome Analysis Toolkit (GATK, version 4.0.8.1, Broad Institute, Cambridge, MA, USA) (https://software.broadinstitute.org/gatk/best-practices/, accessed on 5 August 2022), and SnpEff (version v4_0) (http://pcingola.github.io/SnpEff/, accessed on 5 August 2022) [38]. While the original pipeline had a step for excluding duplicated reads using Picard (version 2.13.2) (https://broadinstitute.github.io/picard/, accessed on 5 August 2022), this step was not included in this study.

Briefly, quality control was performed on raw reads using QCleaner, and low-quality bases (<20 phred scores) were removed. Additionally, reads were removed if 80% of their nucleotides had a quality value <20, if they had sequences of over five unknown nucleotides, if they had <32 base length sequences, or if they did not have a mate-pair. Finally, only high-quality sequences were selected.

BWA was used to align the reads using default parameters to the horse reference genome sequence EquCab3.0 assembly from GenBank (GCA_002863925.1). Alignments were converted from the sequence alignment/map (SAM) format to sorted and indexed binary alignment/map (BAM) files using SAMtools (version 1.8) (http://www.htslib.org/, accessed on 5 August 2022).

GATK was used to detect SNVs and INDELs using the default parameters, with the exception of: −minIndelFrac = 2.0. SNVs, and INDELs were annotated using SnpEff. In this analysis, the GATK VariantFiltration program was not used. The obtained variant data and their depth information in the 52 targeted gene regions were extracted from each VCF file and BAM file and then combined as one file using GENOMINEE (Amelieff Co.). Mapped reads and detected variants were visualized using Integrative Genomics Viewer (IGV, version 2.3.97, Broad Institute).

SNVs were genotyped by collecting variant information (REF-allele, ALT-allele, and their depth) using a BED file in which SNV locations were listed. As HomoREF (REF/REF) was not listed in the VCF file, the depth information of HomoREF was collected from the BAM file using GENOMINEE (Amelieff Co.). REF/REF is HomoREF with sufficient depth information (depth > 50).

## 3. Results and Discussion

### 3.1. Extraction of Genomic DNA from Hair Roots and Blood Samples

All thoroughbreds are parent-verified as part of the registration process. This would also be a suitable time to perform the gene-editing test, as a condition of registration is an unmodified genome. Currently, hair roots or blood are the routine sample types collected for parentage testing of thoroughbreds. Amplicon sequencing using NGS generally requires purified genomic DNA (e.g., 4 ng/μL × 5 μL) as the template DNA, and DNA extracted from blood is known to satisfy library preparation quality and quantity requirements for NGS (Table 2). However, the method for extracting purified genomic DNA required for amplicon sequencing from hair roots has not been properly evaluated. Here, we detail a DNA extraction method from hair roots sufficient for amplicon sequencing.

Genomic DNA was extracted from five and ten hair roots of 11 horses that were less than one-year-old or over two-years-old. The mean concentrations of DNA extracted are shown in Table 2. DNA extracts from either five or ten hair roots did not reach 4 ng/μL, the required quantity for library preparation for NGS. The genomic DNA was concentrated under reduced pressure to obtain amounts of DNA suitable for NGS analysis. This indicates that a gene-editing test could be performed using the same sample collected for the standard parentage test. In a previous study, an average of 8.5 ng/μL (200 μL) of genomic DNA was extracted from 15 hair roots of horses older than one-year-old [39]. However, to streamline the testing of several samples, it may be better to use a small number of hair roots (i.e., five hair roots) to minimize the time spent cutting samples.

### 3.2. Construction of Genome Edited Cells

**Figure 1:**
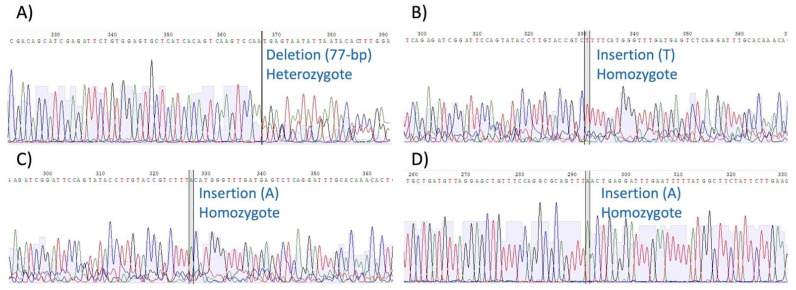
Sanger sequencing detection of variants introduced using CRISPR/Cas9 into the horse *myostatin* gene. (**A**) Edited cell_1 (chr18: 66605476-66605554, exon3-intron2, 77-bp deletion, heterozygote); (**B**) Edited cell_2 (chr18: 66607750, exon2, 1-bp insertion, homozygote); (**C**) Edited cell_3 (chr18: 66607753, exon2, 1-bp insertion, homozygote); (**D**) Edited cell_4 (chr18: 66609936, exon1, 1-bp insertion, homozygote).

Four different genome-edits targeting the *MSTN* gene were introduced into horse fibroblasts, as shown in Table 3. By designing a gRNA complimentary to sequence in exon 3, a heterozygous edited cell lacking 77 bp at the end of intron 2 and start of exon 3 was constructed (Figure 1A, Edited cell_1). Similarly, gRNAs were designed to target two areas in exon 2, and one in exon 1, resulting in homozygous edited cells with a 1-bp insertion in either exon 2 or 1 (Figure 1B–D, Edited cell_2, 3, and 4). The edited sites were all within the designed gRNA sequences.

One of the simplest types of edits to achieve using CRISPR/Cas9 is insertion of a single nucleotide on both DNA strands, creating a novel homozygous alternative version of a gene (homoALT) [40]. For this reason, three of the four edited cells were constructed as such in this study.

The *MSTN* gene is a negative regulator of muscle growth, and animals (including humans) lacking functional myostatin protein have above-average muscle mass [41,42]. Consequently, disruption of this gene has been the target of genome editing in many animals, including the horse [2,12,43]. In both racing whippets and thoroughbred racehorses, naturally occurring variants of the *MSTN* gene affect racing performance [44,45]. In thoroughbred horses, where this phenomenon is well-characterized, the variant alters the amount and composition of muscle fiber types [46,47]. Thus, the *MSTN* gene is an obvious target for gene editing in racehorses, and it was important to show that our screening method would be able to detect *MSTN* gene editing.

### 3.3. Genes Targeted for the Gene-Editing Test and Sequencing Summary

In this study, we targeted 52 genes containing 481 exons (as shown in Table 1) to screen as likely targets for the gene-editing detection test. In total, 1012 regions, including the SNVs incorporated for parent verification described below, were amplified using standard Illumina library preparation steps. The designed primer sequences are not disclosed as this test will be used for gene-doping control.

To validate the method, 120 samples, including 27 duplicate samples, were used (Table 4). Most of the genome-edited cell lines constructed in this study did not grow well, resulting in only a small number of edited cells being developed. To address this, the genomic DNA obtained from cultured edited cells was subjected to whole-genome amplification (WGA) to obtain enough DNA to achieve successful library preparation. Only the Edited Cell_1 line grew enough cells to allow for sufficient genomic DNA to be extracted for library preparation without WGA. It is not known whether this is because the edit made to Edited Cell_1 resulted in a heterozygous change rather than a homozygous change.

Details of the NGS output for the 120 samples are described in Table 5. On average, 1299 SNVs and 94 INDELs were detected in each sample when mapping to all chromosomes without filtering for quality, although these numbers varied widely across the group. A sufficient amount of FASTQ data and mapped reads were obtained from hair roots, blood (with the exception of one sample), and genome-edited cells both with and without WGA and purification. The sample with the smallest amount of FASTQ data was derived from blood that should be most suitable for NGS. No high-quality variant calls were made in the subsequent analysis, and it was assumed an error occurred in the library preparation of this sample.

**Figure 2:**
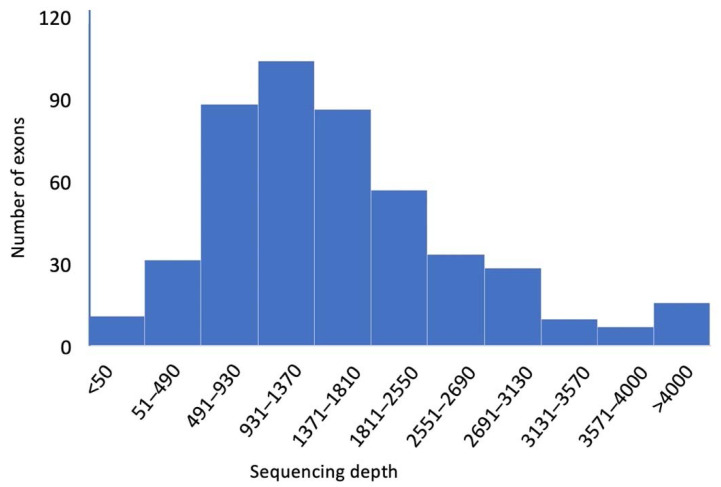
Depth of sequencing coverage of 481 exons in 52 genes. While the coverage varied from exon to exon, over 97% (470 exons) were covered by more than 50 sequence-reads.

Of the 481 targeted exons, 470 (97.7%) had greater than 50× read coverage, 478 exons (99.4%) were covered by more than 20 reads, and all but one exon (99.8%) averaged more than 10× coverage. The variation in coverage was attributed to variable PCR amplification efficiency of each primer (Figure 2). Whilst the coverage obtained in this study was sufficient to be fit for purpose, future iterations of this test could address this by altering the design of primers targeted at exons that showed low coverage in this instance.

### 3.4. Detection of Artificial Mutations in the MSTN Gene Region

**Figure 3:**
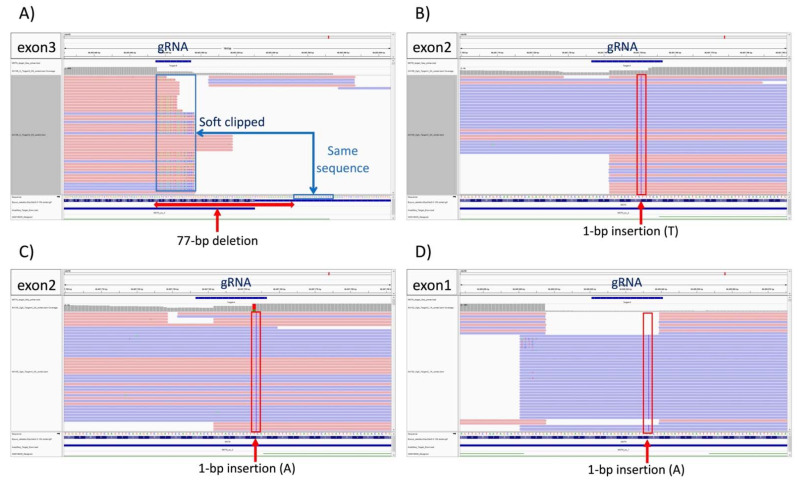
Next-generation sequencing detection of variants introduced using CRISPR/Cas9 into the horse *myostatin* gene. Each variant was visualized using the Integrative Genomics Viewer. (**A**) Edited cell_1 (chr18: 66605476-66605554, exon3-intron2, 77-bp deletion, heterozygote, sequence-reads for the allele without the 77-bp deletion were detected but are out of the frame in the figure); (**B**) Edited cell_2 (chr18: 66607750, exon2, 1-bp insertion, homozygote); (**C**) Edited cell_3 (chr18: 66607753, exon2, 1-bp insertion, homozygote); (**D**) Edited cell_4 (chr18: 66609936, exon1, 1-bp insertion, homozygote).

Using NGS and the analysis pipeline on the 120 samples, the 1-bp insertions (homoALT) introduced into Edited cells_2, 3, and 4 were easily detected (Figure 3B–D). However, the heterozygous 77-bp deletion (REF/ALT) introduced into Edited cell_1 was not detected (Figure 3A). The 77-bp deletion incorporated parts of exon 3 and intron 2 and was located 22-bp from the end of the PCR amplicon. The ends of the amplicon were excluded from variable calling by GATK as this program performs a soft clip at the ends of reads, assuming this region is index or library sequence. Thus, large deletions near the ends of PCR amplicons will be difficult to detect using the analysis pipeline developed in this study.

However, all three homozygous, alternative, single-base-insertion alleles generated in the edited cells were detected by the proposed NGS sequencing and pipeline. CRISPR/Cas9 is particularly efficient at inserting single nucleotides on both DNA strands, creating a novel, homozygous, alternative version of a gene (homoALT) [40]. A single base insertion within a coding region will create a frameshift mutation that (depending on the position of the insertion within the coding sequence) is very likely to be destructive to the function of the mature protein. It is thus important that any gene-editing detection method will be able to detect these small but disruptive edits of gene sequence and function.

The use of CRISPR/Cas9 is not always 100% efficient and may introduce new alleles in the form of compound heterozygotes (ALT1/ALT2) [48]. Based on the results of this study, these should be detected by comparison with the reference sequence using the methods described. Therefore, artificially introduced homozygous alternative and compound heterozygous sequence changes should be identifiable when screening for gene-editing tests.

### 3.5. Establishment of Criteria for Detecting Artificially Edited Variants

An important part of screening for prohibited gene editing is to establish criteria to identify the most likely candidate genome-edited variants (i.e., homoALT or ALT1/ALT2). These should differentiate between naturally occurring variants and those that are more likely to have been artificially introduced.

In this study, a total of 575 variable loci (492 SNVs and 83 INDELs) were detected in the targeted genes in the validation sample set of 120 samples, which included the gene-edited cells. Homozygous INDELs are likely to be preferred by someone wanting to simply knock out the function of a gene. Of the coding INDELs identified, ten were homoALTs and one was a compound heterozygous ALT1/ALT2. The remaining INDELs were heterozygous.

Among the INDELs, four homoALT mutations were excluded as candidates for gene editing because the particular variants had previously been reported in whole-genome sequence data from 101 thoroughbred horses [49]. One compound heterozygous INDEL (ALT1/ALT2) had also been previously observed in the thoroughbred population. Of the remaining six loci, three were excluded as gene-editing candidates because they were covered by extremely low sequence depth in the detected individuals. The remaining three loci were the specific homoALT single-nucleotide insertions introduced into the genome-edited cells (Edited cell_2, 3, and 4), which showed sufficient sequence depth.

Based on the above data, we proposed the following filtering criteria for identifying artificial editing candidates:

(1) Selection of homoALT or ALT1/ALT2 caused by INDELs as artificial editing candidates;
(2) Exclude variants that already exist in the thoroughbred population;
(3) Exclude variants in regions with low sequence depth.

### 3.6. Casework Example

The gene-editing test and detection criteria established in this study were applied to 120 new samples (the casework sample set) collected for the standard parent verification process, which forms an important part of the registration of thoroughbreds in their Stud Book. A total of 508 variants were detected in this group of 120 samples. Those most likely to be gene-editing candidates (homoALT or ALT1/ALT2) constituted seven loci, of which six were already documented as present in the current thoroughbred population. The remaining loci had low coverage. Thus, it is assumed no gene editing was observed in the 120 samples using the developed gene-editing screening test under the described conditions.

### 3.7. Construction of a SNV Panel to Detect Parentage in Thoroughbred Horses

To create genetically modified animals, it is necessary to transplant edited eggs into surrogate mothers to carry to term [50,51]. Therefore, a simple way to identify a genetically engineered foal is to compare the foal’s DNA with that of the mare that carried it. Therefore, we developed a SNV panel to confirm mother–child inheritance.

Primers were designed for amplicon sequencing of 147 loci used in the 2021 SNV comparison test run by the International Society for Animal Genetics (ISAG) [34,35]. These primers were included in the panel for the gene-editing test, and library preparation and sequencing by NGS were performed simultaneously. Three loci failed (chr2:134944, chr9:20379456, and chr18:34581176), leaving 144 SNVs genotyped on the 120 samples used for validating the gene-editing test.

Firstly, 29 trios (sire, dam and foal) were genotyped, and the inheritance of the SNVs by the foals investigated. Of the 144 SNV, 24 SNVs showed mis-inheritances among 27 trios (Table 6). The causes of these mis-inheritances were 1) insufficient coverage (i.e., depth < 50) in all or part of the analyzed individuals and 2) different depths of coverage across different alleles. The former is attributed to the difficulty of multiplexing PCR (1012 amplicons), whereas the latter may involve PCR amplification bias. Trio analysis of the remaining 120 SNV confirmed no mis-inheritances (Table 6). Therefore, these 120 SNVs were selected as the SNV parentage panel.

Next, we evaluated the accuracy of the SNVs for detecting mis-inheritance using 1) five pseudo-trios (pseudo-sire, pseudo-dam, foal), 2) five half-pseudo-trios (sire, pseudo-dam, and foal; or pseudo-sire, dam, and foal), and 3) five pseudo parents (sire or dam)-foal (Table 6). Pseudo-trios, half-pseudo-trios, and pseudo-parent-foal were detected by 29.5, 20.4, and 9.8 SNVs on average using the 120 SNV panel. Table 7 shows the allele frequency, heterozygosity (He), and paternity exclusion (PE) values of the 120 SNVs. The combined PE1 and PE2 values with 120 SNVs were 0.9999999998 and 0.999997, respectively. Five new single parent–foal combinations were also evaluated and parentage confirmed using the selected SNV panel.

Based on these results, the panel of 120 SNVs is capable of clearly identifying the nonbiological dams of thoroughbred foals. A similar method has been developed using maternally inherited mitochondrial DNA sequences [52]. Such approaches can indirectly screen for genetically modified animals; however, it is not possible to directly identify the edited sequences. These approaches may also be useful to identify horses that have been produced by somatic cell cloning, another practice that is prohibited in thoroughbreds.

The development of this DNA profile panel is also important to verify the identity of the tested horses. Any horse that is found to be gene-edited will be required to be DNA profiled to ensure the sample was collected from the correct horse. Having the ability to run the identity analysis simultaneously with the gene-editing test enhances the integrity of the sample analysis. Currently, microsatellite analysis is used for thoroughbred parent verification. However, SNVs are being evaluated for this purpose so that once a final panel of SNVs is determined for standard parent verification across all laboratories this should be incorporated into the gene-editing screening process to allow for simultaneous screening and identification.

One of the reasons to use targeted resequencing to identify genome-edited horses instead of whole-genome resequencing is to reduce the cost of such testing. The gene-editing screening test combined with parentage verification using SNVs cost considerably less than WGR, but more than the currently used parentage verification using microsatellites. The two tests combined cost an amount similar to that of parentage verification using SNVs alone. Thus, once SNVs are confirmed for parentage verification, screening thoroughbreds for gene editing at the same time as parentage verification will be cost-efficient. This will benefit all stakeholders including the breeders, owners, racing authorities, and Stud Books in the racing and breeding industries and will also benefit horse welfare by discouraging prohibited and experimental gene editing.

## 4. Conclusions

The purpose of this study was to construct a screening tool using amplicon sequencing to detect genetically modified thoroughbred horses produced by the use of nonhomologous end-joining with CRISPR/Cas9 in embryos. When 52 gene candidates related to racing performance were targeted and sequenced, 97.7% of the target sequences were covered with sufficient sequence reads to use this method. As a proof of principle, four genetically modified cell lines were screened alongside normal samples. The screening test easily identified the cell lines containing homozygous single-nucleotide insertions, the most efficient product of CRISPR/Cas9 editing. Concurrently, the samples were also DNA profiled, which would allow confirmation of identity if evidence of gene editing were uncovered. It is likely in a real-life gene-editing detection scenario that Sanger sequencing may be necessary to confirm the mutation at that site.

The sequencing of PCR amplified amplicons illustrated that it is extremely difficult to uniformly amplify every targeted genomic region, and some regions may not be covered. Further, it will be difficult to differentiate between artificial and natural heterozygous variants. In this situation, further evidence of gene editing being carried out (for example, access to a laboratory with consumables for gene editing) may be required for successful prosecution. As such, identification of artificially introduced heterozygous variants would not be possible with the gene-editing test developed in this study. Nevertheless, this study is still an important step towards being able to identify genetically engineered thoroughbred racehorses.

## Figures and Tables

**Table 1:** Gene name, location, and analyzed exon numbers for the gene-editing test.

Gene Name	Gene Symbol	Chromosome	Exon Number
angiotensin I converting enzyme	*ACE*	11	25
actinin α 2	*ACTN2*	1	21
actinin α 3	*ACTN3*	12	21
activin A receptor type 1B	*ACVR1B*	6	11
adrenoceptor β 2	*ADRB2*	14	1
angiotensinogen	*AGT*	1	4
AKT serine/threonine kinase 1	*AKT1*	24	13
aldehyde dehydrogenase 2 family member	*ALDH2*	8	13
adenosine monophosphate deaminase 1	*AMPD1*	5	16
apolipoprotein A2	*APOA2*	5	3
apolipoprotein A5	*APOA5*	7	1
bradykinin receptor B2	*BDKRB2*	24	2
creatine kinase, M-type	*CKM*	10	8
clock circadian regulator	*CLOCK*	3	20
ciliary neurotrophic factor	*CNTF*	12	2
erythropoietin	*EPO*	13	5
fibroblast growth factor 1	*FGF1*	14	3
fibroblast growth factor 2	*FGF2*	2	3
fibroblast growth factor 7	*FGF7*	1	3
follistatin	*FST*	21	6
FTO α-ketoglutarate dependent dioxygenase	*FTO*	3	10
growth hormone 1	*GH1*	11	4
hypoxia inducible factor 1 subunit α	*HIF1A*	24	15
insulin-like growth factor 1	*IGF1*	28	4
insulin-like growth factor 2	*IGF2*	12	3
Insulin-like growth factor binding protein 3	*IGFBP3*	4	5
interleukin 15 receptor subunit α	*IL15RA*	29	8
interleukin 1 receptor antagonist	*IL1RN*	15	4
interleukin 6	*IL6*	4	5
interleukin 6 receptor	*IL6R*	5	10
lactase	*LCT*	18	17
lipoprotein lipase	*LPL*	2	10
melanocortin 4 receptor	*MC4R*	8	1
myostatin	*MSTN*	18	3
methylenetetrahydrofolate reductase	*MTHFR*	2	12
5-methyltetrahydrofolate-homocysteine methyltransferase	*MTR*	1	33
5-methyltetrahydrofolate-homocysteine methyltransferase reductase	*MTRR*	21	16
neuromedin B	*NMB*	1	3
nitric oxide synthase 3	*NOS3*	4	25
phosphoenolpyruvate carboxykinase 1	*PCK1*	22	9
pyruvate dehydrogenase kinase 4	*PDK4*	4	12
peroxisome proliferator activated receptor α	*PPARA*	28	6
peroxisome proliferator activated receptor delta	*PPARD*	20	6
peroxisome proliferator activated receptor γ	*PPARG*	16	6
PPARG coactivator 1 α	*PPARGC1A*	3	13
solute carrier family 16 member 1	*SLC16A1*	5	4
solute carrier family 30 member 8	*SLC30A8*	9	10
uncoupling protein 2	*UCP2*	7	8
uncoupling protein 3	*UCP3*	7	6
vitamin D receptor	*VDR*	6	8
vascular endothelial growth factor A	*VEGFA*	20	7
zinc finger and AT-hook domain containing	*ZFAT*	9	17

**Table 2:** Mean concentration of genomic DNA extracted from hair roots or blood in 11 horses that were younger than one year or older than two years.

Sample	Age	Number/ Volume	Mean Concentration (ng/μL)	Standard Deviation
Hair root	0-year-old	5 roots	1.25	0.31
Hair root	0-year-old	10 roots	1.63	0.53
Hair root	2-years-old	5 roots	1.67	0.48
Hair root	2-years-old	10 roots	3.03	0.91
Blood	2-years-old	200 μL	47.2	13.5

**Table 3:** Summary of gene-edited cells constructed using CRISPR/Cas9.

Name	Location	Exon/Intron	Genotype	Reference-Allele	Edited-Allele
Edited cell_1	chr18: 66605476-66605554	Exon3-Intron2	Hetero	77-bp insertion	77-bp deletion
Edited cell_2	chr18: 66607750	Exon2	Homo	C	CT
Edited cell_3	chr18: 66607753	Exon2	Homo	T	TA
Edited cell_4	chr18: 66609936	Exon1	Homo	T	TA

**Table 4:** DNA extraction conditions for 120 samples used for validation of the gene-editing test.

Sample	Condition	Number of Samples Used
Hair roots	Extracted/Purified	46
Hair roots	WGA, Extracted/Purified *	5 ***
Blood	Extracted/Purified	50 ****
Edited cell_1	Extracted/Purified	1
Edited cell_1	WGA, Extracted/Purified *	2
Edited cell_1	WGA **	1
Edited cell_2	WGA, Extracted/Purified *	4
Edited cell_2	WGA **	2
Edited cell_3	WGA, Extracted/Purified *	2
Edited cell_3	WGA **	1
Edited cell_4	WGA, Extracted/Purified *	4
Edited cell_4	WGA **	2

*: Amplified genomic DNA amplified was purified using the DNeasy Blood and Tissue Kit. **: Amplified genomic DNA was directly used for library preparation. ***: The five samples were duplicates of samples included in the group of 46. ****: Four of the fifty blood samples were taken from horses that were also typed using 46 hair root samples.

**Table 5:** Summary of sequencing and variant calling in the gene-editing test.

	Mean	Standard Deviation	Maximum	Minimum
FASTQ data size	218,009,344	37,895,950	397,302,674	17,404,419
Mapped reads *	2,075,806	362,131	3,795,577	116,739
SNV **	1299	854	5345	388
INDEL **	94	56	319	30

*: The number of reads mapped after quality check. **: No filtrations.

**Table 6:** Confirmation of inheritance for 144 and 120 SNVs.

	144 SNVs		120 SNVs	
	Consistent	Inconsistent	Consistent	Inconsistent
Trio_1	142	2	120	0
Trio_2	139	5	120	0
Trio_3	142	2	120	0
Trio_4	143	1	120	0
Trio_5	143	1	120	0
Trio_6	143	1	120	0
Trio_7	141	3	120	0
Trio_8	141	3	120	0
Trio_9	143	1	120	0
Trio_10	140	4	120	0
Trio_11	140	4	120	0
Trio_12	141	3	120	0
Trio_13	141	3	120	0
Trio_14	141	3	120	0
Trio_15	143	1	120	0
Trio_16	141	3	120	0
Trio_17	144	0	120	0
Trio_18	140	4	120	0
Trio_19	141	3	120	0
Trio_20	143	1	120	0
Trio_21	144	0	120	0
Trio_22	142	2	120	0
Trio_23	142	2	120	0
Trio_24	142	2	120	0
Trio_25	142	2	120	0
Trio_26	142	2	120	0
Trio_27	141	3	120	0
Trio_28	141	3	120	0
Trio_29	141	3	120	0
Pseudo-Trio_1	108	36	91	29
Pseudo-Trio_2	110	34	88	32
Pseudo-Trio_3	112	32	91	29
Pseudo-Trio_4	111	33	93	27
Pseudo-Trio_5	108	36	90	30
Half-pseudo-Trio_1	123	21	102	18
Half-pseudo-Trio_2	110	34	95	25
Half-pseudo-Trio_3	119	25	99	21
Half-pseudo-Trio_4	116	28	97	23
Half-pseudo-Trio_5	126	18	105	15
Parent-Foal_1	143	1	120	0
Parent-Foal_2	144	0	120	0
Parent-Foal_3	142	2	120	0
Parent-Foal_4	143	1	120	0
Parent-Foal_5	144	0	120	0
Pseudo-parent-Foal_1	132	12	109	11
Pseudo-parent-Foal_2	128	16	108	12
Pseudo-parent-Foal_3	131	13	110	10
Pseudo-parent-Foal_4	132	12	110	10
Pseudo-parent-Foal_5	136	8	114	6

**Table 7:** Statistical summary of 120 SNVs in the gene-editing test.

SNV ID	Chromosome	Location	REF-allele	ALT-allele	REF frequency	He	PE1	PE2
BIEC2-11336	1	24061861	C	T	0.582	0.487	0.184	0.118
BIEC2-23891	1	58467370	T	C	0.847	0.259	0.113	0.034
BIEC2-34560	1	80153039	T	C	0.520	0.499	0.187	0.125
BIEC2-34987	1	81012651	T	G	0.724	0.399	0.160	0.080
BIEC42118	1	102064366	C	T	0.255	0.380	0.154	0.072
BIEC2-60186	1	138352227	A	C	0.755	0.370	0.151	0.068
BIEC2-65145	1	153834277	C	T	0.635	0.463	0.178	0.107
BIEC2-78523	1	166558525	G	A	0.592	0.483	0.183	0.117
BIEC2-459311	2	17808463	T	C	0.740	0.385	0.156	0.074
BIEC2-476920	2	46942336	C	A	0.633	0.465	0.178	0.108
BIEC2-491394	2	78433836	A	G	0.571	0.490	0.185	0.120
BIEC2-502451	2	98905614	A	G	0.673	0.440	0.172	0.097
BIEC420894	2	120778620	T	C	0.653	0.453	0.175	0.103
BIEC2-777914	3	38956274	T	C	0.622	0.470	0.180	0.110
BIEC672139	3	86457199	T	C	0.694	0.425	0.167	0.090
BIEC2-798927	3	88082655	A	G	0.633	0.465	0.178	0.108
BIEC2-799664	3	90095202	C	A	0.646	0.457	0.176	0.105
BIEC2-800511	3	91766696	T	C	0.235	0.359	0.147	0.065
BIEC2-806771	3	101540587	G	A	0.816	0.300	0.127	0.045
BIEC681989	3	104518656	C	T	0.592	0.483	0.183	0.117
BIEC2-811886	3	119599447	G	A	0.327	0.440	0.172	0.097
BIEC707898	4	178566	T	C	0.729	0.395	0.158	0.078
BIEC2-853347	4	21336785	G	A	0.602	0.479	0.182	0.115
BIEC2-908630	5	45880924	C	T	0.531	0.498	0.187	0.124
BIEC2-910827	5	53722932	T	G	0.604	0.478	0.182	0.114
BIEC2-914714	5	63272427	T	C	0.378	0.470	0.180	0.110
BIEC2-919835	5	73509875	G	T	0.724	0.399	0.160	0.080
BIEC2-946446	6	32343233	A	G	0.704	0.417	0.165	0.087
BIEC798010	6	45478480	A	G	0.398	0.479	0.182	0.115
BIEC811791	6	76708313	G	T	0.622	0.470	0.180	0.110
BIEC819385	7	5215592	A	C	0.480	0.499	0.187	0.125
BIEC2-1007607	7	80202186	C	T	0.520	0.499	0.187	0.125
BIEC870244	8	17383321	G	C	0.653	0.453	0.175	0.103
BIEC871916	8	20028234	A	T	0.551	0.495	0.186	0.122
BIEC2-1052417	8	57040628	C	T	0.714	0.408	0.162	0.083
BIEC2-1066033	8	94179121	G	T	0.520	0.499	0.187	0.125
BIEC2-1066179	8	95184881	G	A	0.735	0.390	0.157	0.076
BIEC2-1080866	9	25656223	T	C	0.551	0.495	0.186	0.122
BIEC915102	9	27114849	C	T	0.500	0.500	0.188	0.125
BIEC86281	10	922571	C	T	0.439	0.493	0.186	0.121
BIEC2-95522	10	4751573	A	G	0.531	0.498	0.187	0.124
BIEC2-97679	10	7701310	G	A	0.643	0.459	0.177	0.105
BIEC2-119640	10	44453287	T	C	0.673	0.440	0.172	0.097
BIEC2-121102	10	48342330	A	G	0.735	0.390	0.157	0.076
BIEC2-123002	10	52573273	T	C	0.867	0.230	0.102	0.026
BIEC2-126732	10	60049178	A	G	0.408	0.483	0.183	0.117
BIEC119158	10	65321527	A	T	0.582	0.487	0.184	0.118
BIEC2-136591	11	7641341	T	C	0.510	0.500	0.187	0.125
BIEC136821	11	29398313	A	G	0.592	0.483	0.183	0.117
BIEC2-162245	11	57232386	C	T	0.653	0.453	0.175	0.103
BIEC154171	12	3878382	G	T	0.337	0.447	0.173	0.100
BIEC155175	12	5186277	C	T	0.698	0.422	0.166	0.089
BIEC2-189666	12	22012560	G	A	0.551	0.495	0.186	0.122
BIEC2-214346	13	19184556	G	A	0.490	0.500	0.187	0.125
BIEC180122	13	35676903	A	G	0.663	0.447	0.173	0.100
BIEC183067	14	4666366	A	G	0.520	0.499	0.187	0.125
BIEC2-245923	14	14775426	C	T	0.704	0.417	0.165	0.087
BIEC204022	14	64551633	A	G	0.813	0.305	0.129	0.046
BIEC2-263616	14	69988182	C	A	0.480	0.499	0.187	0.125
BIEC2-270795	14	83088387	C	T	0.418	0.487	0.184	0.118
BIEC2-278532	15	1201270	A	G	0.776	0.348	0.144	0.061
BIEC247284	15	48661214	G	A	0.633	0.465	0.178	0.108
BIEC2-310269	15	52664101	T	C	0.406	0.482	0.183	0.116
BIEC2-326637	16	1496069	A	G	0.622	0.470	0.180	0.110
BIEC266761	16	3860430	C	T	0.571	0.490	0.185	0.120
BIEC2-328954	16	8598011	A	G	0.398	0.479	0.182	0.115
BIEC2-336269	16	27983909	G	A	0.235	0.359	0.147	0.065
BIEC2-340595	16	36915439	A	G	0.408	0.483	0.183	0.117
BIEC2-342881	16	42323485	C	T	0.602	0.479	0.182	0.115
BIEC2-358061	16	71914513	C	T	0.296	0.417	0.165	0.087
BIEC2-364741	16	83054140	G	A	0.684	0.433	0.169	0.094
BIEC2-364945	16	84257390	C	T	0.541	0.497	0.187	0.123
BIEC2-369699	17	17649634	G	A	0.510	0.500	0.187	0.125
BIEC2-374571	17	27036258	A	G	0.653	0.453	0.175	0.103
BIEC322734	17	78660174	A	G	0.480	0.499	0.187	0.125
BIEC2-390920	18	2190684	A	C	0.745	0.380	0.154	0.072
BIEC324530	18	11431365	C	T	0.633	0.465	0.178	0.108
BIEC2-407206	18	17621415	A	G	0.429	0.490	0.185	0.120
BIEC2-415315	18	56944269	G	A	0.510	0.500	0.187	0.125
BIEC2-430577	19	19611080	A	G	0.296	0.417	0.165	0.087
BIEC2-439060	19	42595105	C	T	0.688	0.430	0.169	0.092
BIEC2-442892	19	48488636	T	C	0.622	0.470	0.180	0.110
BIEC431445	20	16383556	T	C	0.281	0.404	0.161	0.082
BIEC2-530788	20	33600979	T	C	0.480	0.499	0.187	0.125
BIEC2-532106	20	40481673	C	T	0.571	0.490	0.185	0.120
BIEC2-535766	20	49037728	T	C	0.551	0.495	0.186	0.122
BIEC2-575436	22	2011040	C	T	0.582	0.487	0.184	0.118
BIEC2-584911	22	19064667	A	G	0.398	0.479	0.182	0.115
BIEC493879	22	30797481	C	T	0.296	0.417	0.165	0.087
BIEC499860	22	40978756	T	G	0.551	0.495	0.186	0.122
BIEC2-606469	23	4277770	G	A	0.612	0.475	0.181	0.113
BIEC508410	23	16413178	C	G	0.542	0.497	0.187	0.123
BIEC2-618284	23	19540553	G	T	0.750	0.375	0.152	0.070
BIEC523483	24	12615461	T	C	0.229	0.353	0.145	0.062
BIEC2-636987	24	17444264	C	T	0.694	0.425	0.167	0.090
BIEC531275	24	27556162	C	T	0.296	0.417	0.165	0.087
BIEC532060	24	28656489	A	G	0.612	0.475	0.181	0.113
BIEC541693	24	45741983	C	T	0.694	0.425	0.167	0.090
BIEC555737	25	12340280	G	A	0.635	0.463	0.178	0.107
BIEC554813	25	14083078	C	G	0.347	0.453	0.175	0.103
BIEC581695	26	1937434	G	A	0.469	0.498	0.187	0.124
BIEC571705	26	19267701	T	C	0.561	0.493	0.186	0.121
BIEC2-692543	26	30370457	G	A	0.684	0.433	0.169	0.094
BIEC562465	26	42126216	A	G	0.265	0.390	0.157	0.076
BIEC2-708018	27	18560786	C	T	0.724	0.399	0.160	0.080
BIEC590986	27	19761701	T	A	0.663	0.447	0.173	0.100
BIEC2-721061	27	38093323	T	C	0.367	0.465	0.178	0.108
BIEC604433	27	39476969	G	T	0.173	0.287	0.123	0.041
BIEC2-725322	28	5600796	C	T	0.604	0.478	0.182	0.114
BIEC607490	28	5913256	G	T	0.500	0.500	0.188	0.125
BIEC2-734103	28	21956151	C	T	0.439	0.493	0.186	0.121
BIEC617070	28	23842936	T	C	0.704	0.417	0.165	0.087
BIEC2-737704	28	30865784	G	T	0.418	0.487	0.184	0.118
BIEC633181	29	13672303	G	C	0.327	0.440	0.172	0.097
BIEC633979	29	15765096	T	C	0.357	0.459	0.177	0.105
BIEC2-761851	29	29285909	T	G	0.867	0.230	0.102	0.026
BIEC2-816793	30	7757606	T	G	0.531	0.498	0.187	0.124
BIEC686800	30	12032090	T	G	0.153	0.259	0.113	0.034
BIEC696480	31	1930525	T	C	0.490	0.500	0.187	0.125
BIEC2-838630	31	16325150	C	T	0.571	0.490	0.185	0.120

## Data Availability

The primers used have not been listed, as this may prevent their use in actual gene-doping tests. Sequence information of the primers will be provided through a confidentiality agreement with the corresponding author.
